# Combination of radiofrequency ablation and intramedullary nailing for the treatment of femoral metastases: single-center, retrospective observational study

**DOI:** 10.1186/s12957-025-04091-8

**Published:** 2025-11-10

**Authors:** Nokitaka Setsu, Suguru Fukushima, Nobuhiko Yokoyama, Kenji Shinozaki, Mikako Jinnouchi, Takatoshi Fujishita, Ai Nio, Nobuki Furubayashi, Rie Sugimoto

**Affiliations:** 1 Department of Orthopedic Surgery, NHO Kyushu Cancer Center, 3-1-1 Notame, Minami-ku, Fukuoka, 811-1395 Japan; 2 Department of Diagnostic Imaging and Nuclear Medicine, NHO Kyushu Cancer Center, 3-1-1 Notame, Minami-ku, Fukuoka, Japan; 3 Department of Thoracic Oncology, NHO Kyushu Cancer Center, 3-1-1 Notame, Minami-ku, Fukuoka, Japan; 4 Department of Gynecology, NHO Kyushu Cancer Center, 3-1-1 Notame, Minami-ku, Fukuoka, Japan; 5 Department of Urology, NHO Kyushu Cancer Center, 3-1-1 Notame, Minami-ku, Fukuoka, Japan; 6 Department of Hepato-Biliary-Pancreatology, NHO Kyushu Cancer Center, 3-1-1 Notame, Minami-ku, Fukuoka, Japan

**Keywords:** Radiofrequency ablation, Intramedullary nailing, Bone metastases, Impending fracture, Femur

## Abstract

**Background:**

Radiofrequency ablation (RFA) has gained attention as a palliative treatment for bone metastases, providing pain relief and local tumor control. While its use for axial lesions is well documented, its application in long bones remains limited due to concerns about post-ablation fractures. These risks may be mitigated through the combination of RFA with prophylactic intramedullary nailing (IMN).

**Methods:**

Five consecutive patients with femoral metastases who underwent combined RFA and IMN, performed either as a single-stage procedure under intraoperative fluoroscopic guidance or as a two-stage procedure involving CT-guided RFA followed by IMN, were included. Pain relief, function, radiographic response, histology, and complications were retrospectively assessed.

**Results:**

All patients experienced early pain relief and regained mobility. The mean intraoperative blood loss was 48 mL, which was statistically significantly lower than that in the historical control cases (*n* = 8; unpaired two-tailed t-test). At final follow-up, one lesion showed slight progression, three remained stable, and one decreased in size. Complications included one case of nonunion requiring revision surgery and one second-degree skin burn related to electrode pad placement. While immediate pain relief was remarkable, the independent midterm effect of RFA was difficult to determine, as IMN fixation itself provides substantial analgesia and most patients also received postoperative radiotherapy. No additive adverse effects were observed when radiotherapy was combined with RFA.

**Conclusion:**

The combination of RFA and IMN appears to be a feasible and safe minimally invasive option for achieving local tumor control and restoring function in femoral metastases. Potential candidates include those with impending fractures preserving cortical continuity, avulsion fractures of the lesser trochanter, hypervascular tumors, or radiotherapy-resistant lesions. This approach may serve as a less invasive alternative to extensive resection in carefully selected patients.

## Background

Pathological fractures or impending fractures caused by metastatic bone tumors in long bones including the femur and humerus are commonly treated with intramedullary nailing (IMN), which is considered the standard approach. IMN is a minimally invasive procedure involving the insertion of an implant through several small incisions, stabilizing the fracture site, resulting in pain relief and enabling early weight-bearing. However, concerns remain regarding potential tumor seeding during nail insertion and implant failure in the case of tumor progression. To address local tumor control, palliative radiotherapy is often employed in conjunction, and for patients with a longer expected survival, more invasive treatments, such as tumor resection combined with tumor prosthesis replacement, may be selected [1].

Radiofrequency ablation (RFA) is a local ablative technique that delivers high-frequency electrical currents (~ 450 kHz) through needle-shaped electrodes, generating spherical thermal coagulation. Originally used for hepatocellular carcinoma, its application was expanded in Japan to include malignant bone tumors as of September 2022. The advantages of RFA in treating metastatic bone tumors include rapid pain relief [2, 3], local tumor control [4], and intraoperative bleeding reduction when followed by surgery [5, 6]. In comparison with radiotherapy, RFA offers several benefits: it can be completed within hours, is effective even for radioresistant tumors, and is not limited in the number of repeatable treatments. Furthermore, RFA and radiotherapy can be used in combination [7, 8]. However, RFA also carries risks, including early complications such as thermal injury and transient pain exacerbation, as well as delayed complications such as pathological fractures due to post-ablation osteonecrosis [9, 10]. This complication is particularly concerning in weight-bearing long bones such as the femur, which often necessitates surgical intervention.

Only two previous reports have described the combined use of RFA and IMN for the treatment of metastatic lesions in long bones [5, 6]. While its superiority remains unestablished, this combination is theoretically rational: IMN can compensate for RFA-induced structural weakening, whereas RFA can provide local control, which is a limitation of IMN alone. This approach may enable the application of minimally invasive procedures in patients with longer life expectancy by allowing early postoperative mobilization and pain control. Although previous studies have described the combined use of RFA and IMN for metastatic long bone lesions [5, 6], neither included detailed imaging, histological, or perioperative findings. Therefore, this study provides an integrated evaluation of radiological and pathological responses alongside perioperative outcomes, highlighting the feasibility and therapeutic potential of this combined approach.

## Methods

### Study design

We retrospectively evaluated the efficacy and safety of radiofrequency ablation (RFA) combined with intramedullary nailing (IMN) in patients with impending or pathological fractures of the femur due to metastatic bone tumors treated at our institution between July 2023 and July 2024. In addition to clinical and surgical outcomes, this study evaluated radiological and histopathological changes following RFA.

Historical control cases that underwent IMN alone were used to compare the amount of intraoperative blood loss and operative time. Continuous variables were summarized as mean ± standard deviation (SD). The RFA + IMN group was compared with the historical IMN-alone control group in terms of intraoperative blood loss and operative time. Differences between the two independent groups were assessed using a two-tailed unpaired t-test. Statistical significance was defined as *p* < 0.05. To better convey uncertainty given the small sample size, 95% confidence intervals (CIs) for mean differences were calculated using Welch’s t method (unequal variances) with t critical values and Satterthwaite degrees of freedom.

### Patient population

Patients diagnosed with femoral metastatic bone tumors presenting with either impending or pathological fractures, who were considered appropriate candidates for intramedullary nailing (IMN) based on standard surgical indications, were included. Patients with the following conditions were not considered suitable candidates for this treatment: (1) lesions larger than 6 cm in diameter that were deemed unlikely to be sufficiently ablated by RFA; (2) lesions located in areas where RFA needle insertion was considered technically unfeasible; and (3) cases with severe osteosclerosis in which needle insertion was expected to be difficult.

The introduction of RFA for the treatment of bone tumors was approved by the institutional clinical ethics committee. A multidisciplinary cancer board reviewed all the cases and determined treatment strategies before intervention. Informed consent was obtained from all patients before treatment.

To select historical controls, we reviewed 22 consecutive cases of unilateral IMN performed between June 2021 and May 2023. Among these, 3 cases of fragility fractures, 2 atypical femoral fractures, 6 cases with bone metastases larger than 6 cm, and 3 cases with extensive sclerotic lesions were excluded because they were considered unsuitable for RFA based on the criteria described above.

### RFA procedure

RFA was performed using the Cool-tip™ RFA System E Series (Covidien Japan) in accordance with the guidelines and protocols provided by the Japanese Society of Interventional Radiology. An electrode with a 3-cm exposed tip was used in all cases. The number of ablation cycles was adjusted according to lesion size and configuration, assuming a spherical ablation zone of approximately 3 cm in diameter. All procedures were performed in collaboration with interventional radiologists.

### Surgical approach and postoperative management

The choice between single-stage and two-stage treatment depended on the RFA guidance method. When CT-guided RFA was selected, the procedure was performed in an angiography suite, followed by IMN. When RFA was performed through the femoral medullary canal under fluoroscopic guidance, IMN was conducted during the same session as a single-stage procedure. Postoperative management included standard pain control, early mobilization, and periodic clinical and radiographic assessments to monitor healing and detect complications.

## Results

### Patient overview

Five patients underwent combined treatment with RFA and IMN (Table 1). One patient (Case 1) experienced pain recurrence and tumor progression in the femur five months after palliative radiotherapy, whereas the remaining four received postoperative radiation therapy. Two patients underwent two-stage procedures: the IMN procedure was carried out 17 days after RFA in Case 1, and 6 days after RFA in Case 2.

**Table 1:** Patient profiles, therapeutic procedures, and complications

Case	Primary	Age	Sex	Location	Size (cm)	Staged Procedure	Ablation time	Initial resistance	Final Temp.	Operative bleeding	Complication	RT history	Post-op RT	Other adjuvant
1	Lung	70	F	Tr	3	Two	15’	78 Ω	60 ℃	20 ml	Skin burn	+	-	-
				IFx*			7’	78 Ω	75 ℃					
2	Uterine	74	F	Tr	4.5	Two	NA	53 Ω	> 60 ℃	20 ml	-	-	+	Partial curettage
				IFx			7’40”	46 Ω	> 60 ℃					
							9’47”	52 Ω	> 60 ℃					
3	Lung	80	M	Tr	4	Single	5’	108 Ω	89 ℃	50 ml	-	-	+	-
				IFx			2’05”	255 Ω	63 ℃					
							4’	112 Ω	93 ℃					
4	Lung	70	F	SubTr	6	Single	15’	117 Ω	81 ℃	50 ml	Nonunion	-	+	-
				PFx			15’	112 Ω	75 ℃					
							15’	120 Ω	100 ℃					
5	Kidney	71	F	Tr~	4	Single	3’13”	181 Ω	62 ℃	100 ml	-	-	+	Partial curettage
				SubTr	+ 1.5		2’44”	119 Ω	64 ℃					Cementing
					+ 1.5		8’	88 Ω	69 ℃					
				PFx			5’	282 Ω	87 ℃					

*Tr* Trochanteric, *SubTr* Subtrochanteric, *IFx* Impending fracture, *PFx* Pathological complete fracture, *RT* Radiotherapy

*Impending fracture of the shaft accompanied by an avulsion fracture of the lesser trochanter

The eight historical control cases with IMN alone comprised five patients with lung cancer, two with breast cancer, and one with hepatocellular carcinoma; all presented with impending fractures.

### Pain and functional outcomes

Pain levels and functional outcomes during follow-up are summarized in Table 2. In both two-stage cases, including one with avulsion fracture of the lesser trochanter (Case 1), the Numerical Rating Scale (NRS) for movement-related pain improved to 0 within three days after RFA, although the evaluation was performed under non-weight-bearing conditions. Following IMN, pain assessment might have been influenced by surgical site discomfort; however, all patients were ambulatory with manageable pain by postoperative week 1. Pain evaluation was later confounded in Case 1 due to the progression of other bone metastases and in Case 4, who developed nonunion, as described below. Except for these two patients, no treatment site-related complications or symptoms occurred during follow-up.

**Table 2:** Movement-related pain, ambulatory function, and oncological outcome

Case	Pre-treatment	1 day post RFA	3 days post RFA	1 week post-op	4 weeks post-op	12 weeks post-op	Progression of the ablated area	Progression of other bone metastases	Follow-up period	Status
1	NRS 3	NRS 0	NRS 0	NRS 1	NRS 3	NRS 3	Minimal	Enlargement	4 M	DOD
	NWB			Cane	Cane	Walker	progression	Increase		
	Lesser trochanter avulsion					Sacral metastasis				
2	NRS 2	NRS 5	NRS 0	NRS 0	NRS 0	NRS 0	Decreased	NA	8 M	DOD
	NWB			Cane	Cane	Independent		(Solitary)		
3	NRS 0	NA	NA	NRS 5	NRS 0	NRS 0	No change	Increase	5 M	AWD
	NWB			Walker	Cane	Cane				
4	NA	NA	NA	NRS 3	NRS 0	NRS 0	No change	NA	10 M	NED
	Fractured			Crutches	Crutches	Cane		(Solitary)		
				Non-weight bearing	Non-weight bearing					
5	NA	NA	NA	NRS 5	NRS 3	NRS 0	No change	Enlargement	6 M	DOD
	Fractured			Walker	Cane	Cane				

### Imaging and local tumor control

**Fig. 1:**
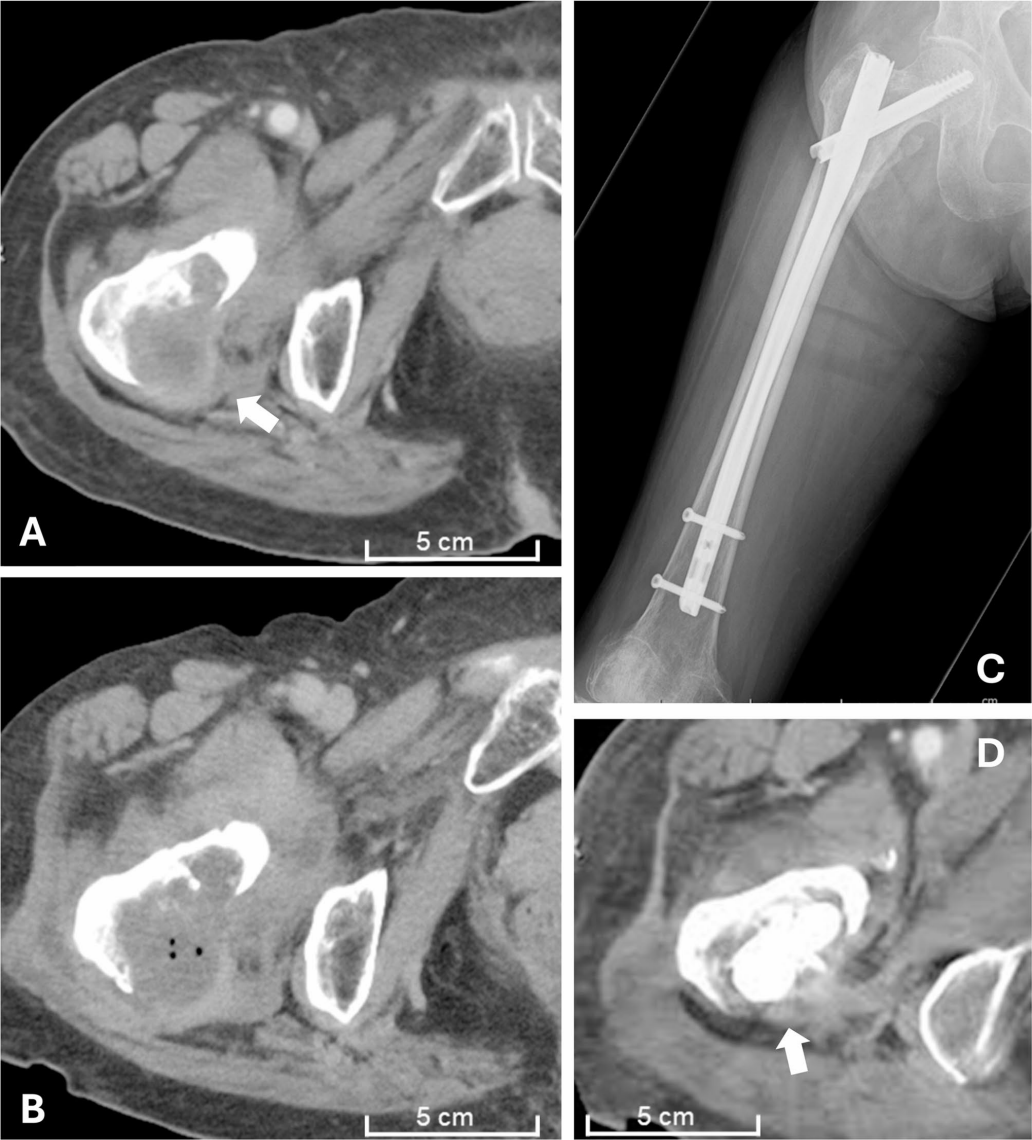
Local control effect of RFA (Case 2). Preablation CT image with contrast enhancement (arrow) (**A**). CT on the day after RFA showing reduced enhancement and central gas formation (**B**). Postoperative radiograph (**C**). Contrast-enhanced CT at 5 months postsurgery showing posterior extraosseous mass reduction without curettage in this area (arrow) (**D**). RFA: Radiofrequency ablation

In both two-stage cases, contrast-enhanced CT performed on the day after RFA showed reduced enhancement at the ablation site (Fig. 1). At the final follow-up, one lesion showed slight progression, three remained stable, and one decreased in size (Table 2). Among the three patients with bone metastases at sites other than the treated femur, disease progression was observed at those locations. Bone formation was not observed at any RFA-treated site. Overall, CT findings suggested a local tumor control effect of RFA, consistent with the reduced enhancement and size stability observed during follow-up.

### PET and bone scintigraphy findings and histological findings

**Fig. 2:**
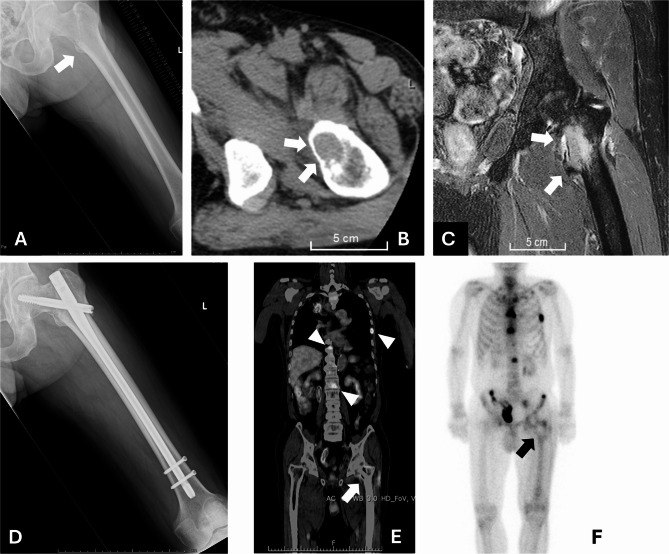
Preservation of bone regenerative potential after RFA (Case 3). Pretreatment radiograph (**A**), pretreatment CT image (**B**), and fat-suppressed T2-weighted image (**C**) showing the metastatic lesion (arrow). Posttreatment radiograph (**D**). PET-CT one month after treatment revealed uptake in multiple metastatic bone lesions (arrowheads) but no uptake at the RFA-ablated site (arrow) (**E**). Bone scintigraphy four months after treatment showing uptake comparable to that of the surrounding cortical bone (arrow) (**F**). RFA: Radiofrequency ablation

In Case 3, postoperative PET revealed no abnormal FDG uptake at the ablated site (Fig. 2), indicating successful tumor inactivation by RFA. Conversely, bone scintigraphy at four months demonstrated preserved tracer uptake, implying maintained osteogenic activity despite tumor ablation. Histopathological examination of intramedullary specimens revealed consistent degenerative changes—including nuclear swelling, chromatin homogenization, and vacuolar degeneration—in all five cases. In the two cases treated in a staged manner, additional inflammatory cell infiltration was noted, suggesting early post-ablation response. In Case 1, sampled 17 days after RFA, only minimal viable tumor cells were identified. In contrast, in the other four cases—including Case 3, which showed no FDG uptake—approximately 20–50% of tumor cells remained viable. Taken together with imaging and clinical findings, histopathological evaluation alone may not reliably reflect the therapeutic effects observed radiologically.

### Complications

Nonunion: One patient (Case 4) developed nonunion with IMN failure at 6 months and required revision surgery with proximal femoral resection and prosthesis replacement. Histological examination of the resected specimen revealed fibrous or necrotic tissue, approximately 60% preservation of osteocytes within the cortical bone, and no evident residual tumor cells, suggesting partial osteonecrosis likely related to excessive ablation temperature.

Burn injury: One patient developed a second-degree burn on the skin of the bilateral medial thighs, probably due to an electrical shortcut between the electrode pad on the contralateral thigh and the RFA needle. Modifying the electrode pad placement to the trunk prevented recurrence.

Intraoperative and procedural tolerance Two: patients reported discomfort during RFA under local anesthesia, which was managed with cooling, analgesics, and sedation. The RFA + IMN group (*n* = 5) was compared with the historical IMN-alone control group (*n* = 8) regarding intraoperative blood loss and operative time (Table 3). The mean intraoperative blood loss was significantly lower in the RFA group (48.0 ± 32.7 mL) than in the historical control group (156.9 ± 88.9 mL; mean difference = − 109 mL, 95% CI [− 187, − 31], *p* = 0.011). The mean operative time was longer in patients who underwent RFA, but the difference was not statistically significant (mean difference 25.8 min; 95% CI [− 31.5, 83.0], *p* = 0.348).

**Table 3:** Operative blood loss and time: IMN with vs. without RFA

	n	Blood loss (ml), mean	SD	*P*-value	Operative time (min), mean	SD	*P*-value
RFA + IMN	5	48.0	32.7	0.011*	131.4	45.3	0.348
IMN control	8	156.9	88.9		105.6	46.2	

*RFA* Radiofrequency Ablation, *IMN* Intramedullary Nailing

*Statistically significant (*p* < 0.05)

## Discussion

When RFA is used for metastatic bone tumors, its primary role remains palliative, aimed at achieving rapid pain relief. However, RFA also offers benefits in terms of local tumor control and intraoperative hemostasis during subsequent surgical procedures. Therefore, combining RFA with IMN fixation may provide multiple synergistic advantages, though further validation is warranted. In this study, the combination demonstrated short- to mid-term safety and effectiveness, particularly for impending fractures.

The RFA needle must be inserted carefully to avoid damaging major vessels and nerves, such as the femoral artery, vein, femoral nerve, and sciatic nerve. A vertical approach to the lesion is preferable for cortical destruction or lesser trochanter avulsion fractures, making CT-guided RFA technically desirable. When intraoperative CT is available, a single-stage procedure may be feasible; otherwise, a two-stage approach—performing RFA under CT guidance, confirming the ablation area with contrast-enhanced CT imaging, and proceeding with IMN—is appropriate. In two-stage procedures, patients should be maintained in strict non-weight-bearing conditions between RFA and IMN to prevent fracture. Conversely, when cortical continuity is preserved and the lesion remains confined within the medullary cavity, accessing the tumor longitudinally via the intramedullary canal under fluoroscopic guidance is more appropriate. In such cases, CT-guided RFA is technically challenging; thus, a single-stage procedure with simultaneous RFA and IMN can be a practical option. A single-stage procedure is also appropriate if direct intraoperative tumor ablation is needed.

In this study, RFA achieved remarkable short-term pain relief in cases where immediate post-ablation assessment was possible. Remarkably, in one patient with an avulsion fracture of the lesser trochanter, marked relief of motion-induced pain was observed, suggesting the utility of RFA for lesser trochanter avulsions in which IMN cannot stabilize. Isolating the specific contribution of RFA to midterm pain relief remains challenging because IMN fixation alone affords substantial pain relief by stabilizing fractures. Previous retrospective studies have also suggested that RFA, when used adjunctively with IMN, tends to provide better pain control than radiotherapy with IMN [6]. However, in the present series, postoperative radiotherapy was performed in most patients, except for those with recurrence after prior radiation therapy. Therefore, it is difficult to evaluate the independent pain relief effect of RFA alone, similar to the challenges in assessing its tumor control effect. Of note, even when postoperative radiotherapy was combined with RFA, no increase in adverse effects was observed. Several reports have described the usefulness of combining RFA and radiotherapy for treating spinal metastases [7, 8]; thus, similar synergistic effects may be expected in long bones such as the femur. At the very least, in cases where radiotherapy is contraindicated, unavailable, or less effective, RFA is a useful adjunctive therapy alongside IMN fixation.

Thermal ablation for tumor control conceptually parallels the pasteurization technique, an established method for autograft recycling that heats bone to 60–65 °C for 30–40 min to eradicate malignant cells while preserving its structural and biological integrity [11]. Nevertheless, as with pasteurized bone, RFA-treated bone may exhibit delayed or incomplete union. Experimental studies have reported that although osteocyte remnants persist after RFA, partial bone necrosis has been observed [12]. In one patient with both PET and bone scintigraphy available after RFA, FDG uptake disappeared while tracer activity on bone scintigraphy persisted, suggesting preserved bone metabolic potential despite effective tumor ablation. In contrast, in the nonunion case, histology revealed residual osteocytes but minimal new bone formation, implying impaired osteoinductive potential likely secondary to thermal denaturation and necrotic matrix formation, possibly related to the higher ablation temperature in this case. Although previous reports suggested that RFA provides superior pain relief in patients with pathological fractures [13, 14], its use in fractured weight-bearing bones such as the femur may increase the risk of delayed healing or implant failure. Based on these considerations, restricting indications to impending fractures with preserved cortical continuity, with cement augmentation when appropriate, and selecting an electrode that limits ablation to the minimally required area appears safer and more predictable. To minimize osteonecrosis and preserve bone-healing potential, we later adopted an ablation strategy mimicking pasteurization conditions by not prioritizing roll-off; instead, ablation was paused at four minutes to confirm temperature, maintaining 60–70 °C for approximately five minutes. Unlike hepatic ablation, bone ablation cannot be monitored ultrasonographically. Moreover, our cooling-electrode system lacked real-time temperature monitoring, requiring intermittent pauses of about 20 s for temperature measurement. Further investigation is warranted to optimize temperature monitoring protocols and ablation duration for safe application in long bones.

Objective evaluation of RFA efficacy remains inherently difficult. At our institution, contrast-enhanced CT is typically obtained on the following day to confirm reduced enhancement at the ablation site. This assessment is feasible in two-stage procedures but is likely hindered by metallic artifacts from intramedullary nails in single-stage cases. Delayed postoperative PET imaging may offer further information on local tumor control. In this series, one patient showed no abnormal FDG uptake at the ablated site, indicating successful inactivation; however, this was coincidental with diagnostic staging and is not practical as a routine post-RFA evaluation considering medical cost. Histopathological assessment after RFA can also yield valuable information. In our cases with available samples, degenerative nuclear changes were observed, although morphologically viable-appearing tumor cells persisted, including cytokeratin-positive cells on immunostaining. Even in the PET-negative case, viable cells were identified histologically. However, in the case that later required resection, no residual tumor cells were identified histologically. These findings suggest that morphological or immunohistochemical viability immediately after RFA does not necessarily indicate true tumor activity, as cellular structure and antigenicity may be transiently preserved. Consequently, treatment efficacy should be evaluated through serial imaging follow-up, focusing on changes in extraosseous tumor size and enhancement. Ultimately, the contribution of RFA to sustained local control should be confirmed in larger, prospective studies.

This study identified one case each of nonunion and thermal skin injury. Previous reports on IMN alone have shown that, when comparing metastatic and non-metastatic femoral fractures, surgical adverse events such as acute anemia (47.9%) occurred significantly more frequently in metastatic fractures, although the reoperation rate did not differ between groups [15]. Another study comparing pathological and impending fractures of the femur reported reoperation in 1 case (3%) among pathological fractures and in 9 cases (7%) among impending fractures, with no significant difference [16]. Among the complications in the present study, the skin burn was a distinctive event associated with RFA, which can be prevented by careful electrode placement and adequate cooling during the procedure. Although one of five patients developed delayed union and implant breakage requiring revision surgery, this frequency may be reduced by limiting the indication to impending fractures or by adding cement augmentation when appropriate. Other than nonunion and thermal skin injury, no other RFA-related complication was observed. IMN combined with RFA resulted in significantly lower intraoperative blood loss compared with IMN-alone control patients, consistent with previous reports [5, 6]. This combined approach demonstrated a clear hemostatic advantage during IMN, suggesting particular suitability for hypervascular metastases such as those from renal or thyroid carcinoma. Although the operative time tended to be longer in the combined treatment group, the difference was not statistically significant. Importantly, all control cases involved impending fractures, whereas two patients in the combined treatment group presented with complete fractures at the time of treatment. This difference likely led to overestimation of operative time due to the additional exposure and reduction steps required for complete fractures, suggesting that the apparent prolongation is unlikely to be clinically meaningful.

Taken together, the combined RFA and IMN technique appeared safe and effective for impending fractures, providing complementary benefits of pain relief, local tumor control, and intraoperative hemostasis. However, in complete pathological fractures, cautious application is warranted because of the potential risk of delayed bone healing or nonunion after ablation. Given the very small sample size (*n* = 5) and retrospective design, these findings should be interpreted as preliminary and hypothesis-generating rather than definitive. In particular, the use of historical controls for comparison of intraoperative blood loss and operative time is subject to potential confounders, such as differences in surgeon or patient comorbidities, thereby limiting the reliability of the comparative analysis. Moreover, the concomitant use of radiotherapy in most patients made it difficult to isolate the independent effects of RFA on local tumor control and pain relief. Future multicenter studies with larger cohorts and prospective designs are warranted to validate these observations and refine patient selection criteria.

## Conclusion

RFA combined with intramedullary nailing for long bone metastases appears to be a minimally invasive and effective strategy for achieving local tumor control while restoring function. Although wide resection with tumor prosthesis reconstruction is often preferred in patients with longer life expectancy, this combined technique may represent a less invasive alternative. In line with previous limited reports, our findings add histological and imaging-based evidence, together with perioperative data, to support the feasibility of this combined approach.

At present, potential indications for this combined therapy include impending fractures with preserved cortical continuity, avulsion of the lesser trochanter, hypervascular lesions, and radioresistant tumors. To ensure effectiveness and safety, we recommend checking the electrode tip temperature approximately four minutes after the start of ablation.

## Data Availability

The datasets used and/or analyzed during the current study are available from the corresponding author upon reasonable request.

## Abbreviations

IMN: Intramedullary nailing
RFA: Radiofrequency ablation
NRS: Numerical Rating Scale
